# MAPK Usage in Periodontal Disease Progression

**DOI:** 10.1155/2012/308943

**Published:** 2012-01-23

**Authors:** Qiyan Li, Michael S. Valerio, Keith L. Kirkwood

**Affiliations:** ^1^Department of Endodontics, Periodontics and Oral Medicine, The First People's Hospital of Yunnan Province, Kunming, Yunnan 650032, China; ^2^Department of Craniofacial Biology and Center for Oral Health Research, Medical University of South Carolina, Charleston, SC 29425, USA

## Abstract

In periodontal disease, host recognition of bacterial constituents, including lipopolysaccharide (LPS), induces p38 MAPK activation and subsequent inflammatory cytokine expression, favoring osteoclastogenesis and increased net bone resorption in the local periodontal environment. In this paper, we discuss evidence that the p38/MAPK-activated protein kinase-2 (MK2) signaling axis is needed for periodontal disease progression: an orally administered p38**α** inhibitor reduced the progression of experimental periodontal bone loss by reducing inflammation and cytokine expression. Subsequently, the significance of p38 signaling was confirmed with RNA interference to attenuate MK2-reduced cytokine expression and LPS-induced alveolar bone loss. MAPK phosphatase-1 (MKP-1), a negative regulator of MAPK activation, was also critical for periodontal disease progression. In MPK-1-deficient mice, p38-sustained activation increased osteoclast formation and bone loss, whereas MKP-1 overexpression dampened p38 signaling and subsequent cytokine expression. Finally, overexpression of the p38/MK2 target RNA-binding tristetraprolin (TTP) decreased mRNA stability of key inflammatory cytokines at the posttranscriptional level, thereby protecting against periodontal inflammation. Collectively, these studies highlight the importance of p38 MAPK signaling in immune cytokine production and periodontal disease progression.

## 1. Innate Immunity and Periodontal Disease

### 1.1. Host-Microbe Interaction

Within the oral cavity exists a biofilm colonized by more than 500 different microbial species, very few of which are actually associated with periodontal disease [1–3]. These periopathogenic gram-negative bacteria contain multiple virulence factors, including lipopolysaccharide (LPS), which can induce the host inflammatory response. In periodontal diseases initiation and progression, such an inflammatory response to bacterial biofilm is exaggerated, resulting in leading to overproduction of inflammatory cytokines that cause gingival inflammation, bleeding, extracellular matrix degradation, bone resorption, and tooth loss [4–6].

Over the past two decades, how host-microbe interactions contribute to both disease initiation and associated tissue destruction have been elucidated. Epidemiological data indicate different intraindividual susceptibilities to periodontal disease, despite the long-term presence of oral biofilm [7–9]. Moreover, increased susceptibility and greater severity of periodontal disease were observed in individuals with impaired immune responses [10, 11]. The most significant development in periodontitis research has been the fundamental role of innate immunity in initiating immune responses and regulating adaptive (antigen-specific) responses [5]. The innate immune response recognizes and responds to all colonizing microbes, both commensal and pathogenic. The modest cytokine response to commensal bacteria stimulation in the periodontium is necessary for priming host immunity and maintaining tissue integrity, and the amplified immune response is induced when the microbial composition of plaque, in which pathogenic bacteria are greatest, changed [12, 13].

**Figure 1:**
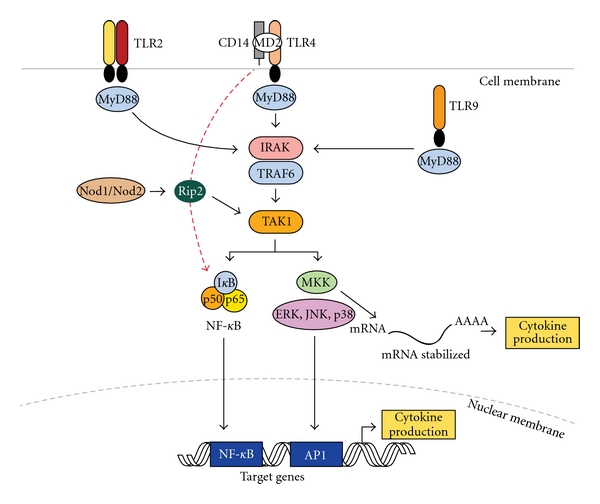
Pattern recognition receptors and innate immune signaling. TLR-2, TLR-4, and TLR-9 are depicted as examples of TLR receptors expressed in cells of the periodontal tissues. Upon ligand binding, all TLRs (except TLR3) recruit adaptor protein MyD88 and activate common upstream activator (IRAK/TRAF6 and TAK1) of NF-*k*B and MAP kinases. TLR-4 may also activate NF-*k*B independent of MyD88 and with delayed kinetics (red dotted line). Nod1/Nod2 are cytosolic PRRs that recognize peptidoglycan fragments of the bacterial wall and may amplify TLR-induced activation of signaling pathways. Activated NF-*k*B and MAP kinases translocate to the nucleus and bind to their motifs (NF-*k*B, AP-1, resp.) in the promoter of target genes (including early-response and inflammatory genes) and induce their transcription into mRNA which will ultimately lead to increased cytokine production. p38 MAP kinase also involved posttranscriptional regulation of pro-inflammatory genes (for example, IL-6, Cox-2) by modulation of mRNA stability in the cytoplasm. (TLR: toll-like receptor, CD14: cluster of differentiation 14 molecule; MD2: myeloid differentiation protein 2; MyD88: myeloid differentiation primary response gene 88; IRAK: interleukin-1 receptor-associated kinase; TRAF6: TNF receptor associated factor 6; TAK1: TGF-beta activated kinase 1; MKK: mitogen-activated protein kinase kinase; ERK: extracellular signal-regulated kinase: JNK: c-Jun N-terminal kinase; AP-1: activator protein-1).

In the current paradigm, Toll-like receptors (TLRs) link the host and microbes and are considered essential for LPS-induced signaling. LPS, one of the main pathogen-associated molecular patterns (PAMPs) of pathogenic bacteria, is recognized by the host through TLRs, resulting in activation of multiple downstream cell signaling cascades [14]. To date, the TLR family includes 13 members, which is consistent with the range of PAMPs expressed by infective microorganisms. These receptors not only recognize various PAMPs and activate innate immune response, but they can also bind to endogenous molecules derived from damaged tissue and contribute to innate inflammation as well as the adaptive immune response [15]. Within the periodontium, innate immunity is comprised of resident immune cells such as monocytes/macrophages, neutrophils, dendritic cells, and nonimmune resident cells such as periodontal fibroblasts and gingival epithelial cells. Accordingly, all of these cell types express various TLRs to identify and respond temporarily to PAMPs [16–18]. In periodontal tissues, TLR2 and TLR4 expression has been positively correlated with disease severity, suggesting that these receptors have an increased capacity to signal and influence downstream cytokine expression [19–21]. All TLRs are single-pass transmembrane proteins containing a common extracellular N-terminal leucine-rich domain and a conserved intracellular C-terminal domain. The N-terminal domain is responsible for the recognition of the ligands and the C-terminal tail is shown to be homologous with the intracellular domain of the interleukin-1 receptor type I, currently designated as the Toll/IL-1 receptor (TIR) domain [22]. The classic intracellular signaling pathways activated by TLR engagement are highly conserved. The TLR-PAMP interaction recruits specific adaptor molecules which then bind the interleukin (IL)-1 receptor associated kinase (IRAK), initiating a chain of signaling transduction. In the TLR pathway, at least four adaptor proteins, including myleloid differentiation primary-response protein 88 (MyD88), TIR domain-containing adaptor-inducing interferon *β* (TRIF), MyD88 adapter-like/TIR domain-containing adaptor protein (Mal/TIRAP), and TRIF-related adaptor molecule (TRAM), contain TIR domains that can be recruited by activated TLRs. Each of these adaptor molecules interact with the various TLRs, an event thought to be responsible for signal transduction branching and significant TLR signaling flexibility by allowing crosstalk with other pathways, including MAP kinase, PKR, and Notch pathways [23–27] (see Figure 1).

Of the TLR signaling pathways, the most studied is the recognition of LPS by a macromolecular complex involving CD14, MD2, and TLR4 [6, 28, 29]. In response to LPS, complex formation triggers MyD88 and, in turn, recruits IRAK and TRAF6. The phosphorylated IRAK/TRAF6 complex then dissociates from the receptor complex to a new complex with transforming growth factor *β*-activated kinase (TAK1) along with TAK-1 and -2 binding proteins (TAB1 and -2) which phosphorylate TAK1. TAK1, in turn, phosphorylates both the inhibitor of nuclear factor *κ*B-(I*κ*B-) kinase complex (IKK complex) and mitogen-activated protein kinase (MAPK) kinases-3 and -6 (MKK3, MKK6) [30]. The IKK complex then phosphorylates I*κ*B, which allows nuclear factor-kappa B (NF-kB) transcription factors (p50/p65) to translocate to the nucleus and bind to the promoter regions of many proinflammatory cytokine and chemokine genes and activate their transcription [31, 32]. Similarly, MKK3/6 can phosphorylate p38 and c-jun N-terminal kinase (JNK) MAPK to activate activator protein-1 (AP-1) transcription factors (TFs) and initiate gene expression. In addition, p38 can phosphorylate RNA-binding proteins, which can stabilize cytokine mRNA and thus amplify cytokine production [33], as depicted in Figure 1.

### 1.2. Genesis of Inflammation in Periodontal Tissues

Periodontal tissue destruction is initiated by pathogens that colonize in the periodontal pocket, a unique microenvironment in the subgingiva that fosters the growth of anaerobic bacteria and spirochetes. These microorganisms produce harmful byproducts and enzymes, such as collagenases and proteases, which degrade the extracellular matrix to produce nutrients for their growth. Resultant tissue damage creates pathways for invasion, followed by irritation and inflammation of host tissues, eliciting a host immune response. This local immune response in gingival tissues involves recruitment of inflammatory cells, generation of cytokines and prostanoids, elaboration of lytic enzymes, and osteoclast activation [34–36].

Within the inflamed periodontium exist various resident immune and nonimmune cells: periodontal ligament cells, fibroblasts, osteoblasts, osteoclasts, neutrophils, antigen-presenting cells such as dendritic cells, macrophages, T cells, and B cells. These resident cell types recognize and interact with bacterial constituents (e.g., cytoplasmic membranes, peptidoglycans, outer membrane proteins, lipopolysaccharide, capsules, and cell-surface fimbriae) to generate proinflammatory mediators, such as IL-1, tumor necrosis factor (TNF)-*α*, IL-6, and prostaglandin E_2_ (PGE_2_) [37–39]. These secreted molecules can recruit more nonresident cells (e.g., neutrophils, macrophages, and lymphocytes) to infiltrate tissues and initiate the innate immune response [40, 41]. They also further induce their own expressions through numerous autocrine or paracrine mechanisms and thus perpetuate and amplify chronic periodontal inflammation. Additionally, cellular components and byproducts of tissue breakdown in the periodontium can be recognized and trigger signal cytokine secretion, possibly creating a self-sustaining positive feedback circuit, leading to loss of tissue function and exacerbated clinical disease. With overexpression of proinflammatory cytokines, other inflammatory enzymes and mediators, such as matrix metalloproteinases (MMPs) and receptor activator of nuclear factor-kB ligand (RANKL), are upregulated to produce irreversible soft and hard tissue damage.

Within periodontal lesions, activated monocytes, macrophages, and fibroblasts all produce cytokines, such as TNF-*α*, L-1*β*, IL-6, and PGE_2_, which are significantly elevated in diseased periodontal sites, compared with healthy or inactive sites [42–48] and have been positively correlated with increased probing depth and attachment loss [49–51]. In the gingival crevicular fluid, elevated IL-6, TNF-*α*, and IL-1*β* were reported in persons afflicted with periodontitis [52, 53]. Elevated IL-6 is higher in recurrent periodontitis cases and increased GCF correlates with gram-negative fimbriae [53–55].

Periodontal disease manifests as an intimate combination of inflammation and bone resorption, leading to the eventual tooth loss. Bone is a dynamic tissue that constantly undergoes a remodeling process in which bone resorption and bone deposition are balanced.When chronic inflammation occurs in bone and other mineralized tissue, this balance is disrupted to favor net bone loss. In periodontitis, alveolar bone resorption occurs when inflammatory mediators in the overlying soft tissues reach a certain threshold at a critical distance from the bone surface and activate pathways leading to bone resorption [56]. 

The RANK/RANK ligand (RANKL)/osteoprotegerin (OPG) system which controls osteoclast development, differentiation, activation, and function, is a key mediator of bone loss in periodontal disease. Through interactions with its cognate receptor RANK on the cell surface of osteoclasts and osteoclast precursors, RANKL stimulates differentiation and maturation of cells from the monocyte/macrophage lineage to form functional osteoclasts and then induces osteoclastogenesis and subsequent bone resorption. OPG, a soluble member of the tumor necrosis family produced by osteoblasts, marrow stromal cells, and other cells, can bind to RANKL as a decoy receptor and inhibit osteoclast differentiation to balance this system, decreasing RANKL functional activity. Thus, the RANKL/OPG ratio eventually determines bone turnover [6, 57, 58]. 

Even though RANKL expression may require different signaling pathways depending on the nature of extracellular stimulation, cell type, and even cell differentiation state, RANKL expression has been shown to increase inflamed periodontal tissues from various cell types, including osteoblastic cells, bone marrow stromal cells, endothelial cells, mononuclear cells, and periodontal ligament fibroblasts [59–63]. More recently, many secreted proinflammatory cytokines and other mediators have been shown to converge and stimulate RANKL in these cell types. Meanwhile, most of these same molecules decreased OPG production. Such molecules include IL-1*β*, TNF-*α*, IL-6, IL-8, IL-11, IL-17, MMPs, and PGE_2_, all of which are upregulated in periodontal tissues. With alveolar bone loss, the periodontal pocket deepens and subgingival plaque biofilm further accumulates. The subgingival microflora becomes more anaerobic and the host response becomes more destructive and chronic.

## 2. Pathobiology of Periodontal Disease Progression

### 2.1. MAPK Cell Signaling

The innate immune system serves as a first defense against invading pathogenic organisms and its interaction with microbial components activates multiple signaling cascades, including MAP kinase pathways. Among the various signaling molecules that regulate inflammatory pathways, kinases are important because their aberrant or upregulated expression significantly contributes to the regulation of inflammatory disease. MAP kinases are highly conserved serine/threonine protein kinases in eukaryotes. MAP kinase BMK-1/ERK5 has been reported to respond to both growth factors and stressful stimuli, contributing to cell cycle progression and proliferation, but three other MAP kinases, p38, c-Jun N-terminal kinases JNK, and extracellular signal-regulated kinases ERK, are the most studied [64].

Different cell stimuli preferentially activate distinct MAP kinases and endow them with different functions. Many growth factor and G protein linked receptors, cell adhesion, phorbol esters, and some oncogenes are linked to activation of the ERK MAP kinases, which are involved in cellular chemotaxis, cell cycle progression and mitogenesis, oncogenic transformation and metastasis, neuronal differentiation and survival, and in processes underlying memory and learning. Inflammatory cytokines (such as IL-1 and TNF-*α*), trophic factor deprivation, and a number of cell stress-inducing factors (such as heat shock, osmotic shock, ultraviolet radiation, and oxygen radicals) preferentially lead to activation of JNK/SAPK and p38 MAP kinases. In all, MAPK signal transduction cascades play a pivotal regulatory role in the biosynthesis of numerous cytokines, chemokines, and other inflammatory mediators that are necessary for the immune system to combat pathogenic infections.

The MAPKs are organized in modules (MAPKKK→MAPKK→MAPK) sequentially activated by a cascade of dual phosphorylation events at tyrosine/threonine residues. Beginning with the activation of upstream MAP kinase kinase kinase (MKKK), MAP kinase kinase (MKK) is further activated by MKKK at two serine residues. MKK in turn activates MAP kinase by phosphorylating the MAPKs at the adjacent threonine and tyrosine residues localized within a conserved activation loop motif, T*x*Y, between the kinase subdomains VII and VIII (where *x* represents glutamate in ERK, proline in JNK/SAPK, and glycine in p38 MAPKs) [65]. Furthermore, MKK-3 and -6 are specific for p38 whereas MKK-4 also activates JNKs. Once activated, the MAPKs can target an array of downstream substrate proteins for phosphorylation, including the “downstream” serine/threonine kinases (which themselves become activated), cytoskeletal elements, cell death regulators, and many nuclear receptors and transcription factors (including AP-1 (homodimer or heterodimer of the proteins c-fos and c-jun), NF-*κ*B, or CAAT-enhancer-binding protein) which facilitate gene transcription. For example, NF-*κ*B can bind to the promoter regions of many proinflammatory cytokine and chemokine genes and activate their transcription. In addition to the regulation of the expression of inflammatory mediators, MAPKs are also implicated in the regulation of reactive oxygen and nitrogen species, which are critical for killing microbes engulfed by phagocytes. MAPKs also regulate gene expression through promoting chromatin remodeling [64] and participate in the transport, stabilization, and translation of cytokine mRNA transcripts that contain AU-rich elements [33]. It is well known that p38 MAPK activates MAP kinase-activated protein kinase (MK)-2 through phosphorylation. MK-2, in turn, inactivates transacting RNA-binding protein tristetraprolin (TTP) by phosphorylation at serine 52 and 178. This phosphorylation sequesters TTP with 14 : 3 : 3 and inhibits the binding of TTP with ARE mRNA. Thus, ARE mRNAs are spared from TTP shuttling to degradation machinery, and TTP-mediated deadenylation and destabilization of ARE-containing transcripts are inhibited, giving mRNAs an opportunity for translation [66–68].

**Figure 2:**
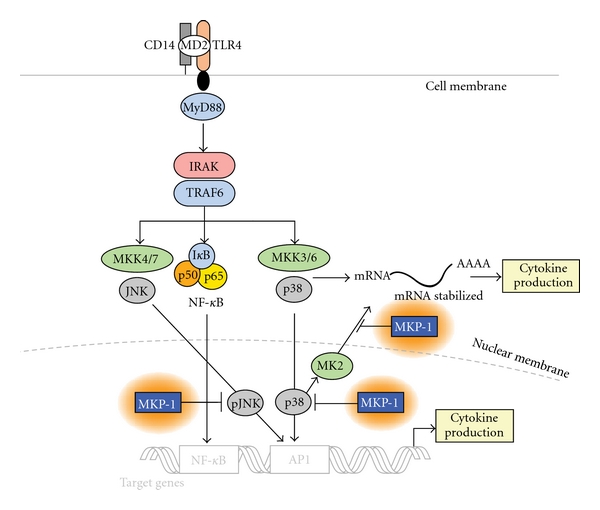
MAPK activation and regulation via MKP-1. Bacterial LPS induces MAPK signaling as described in Figure 1. MAP Kinase Phosphatase-1 (MKP-1) negatively regulates MAPK activation via dephosphorylation of target kinases at multiple points of kinase activation.

Once the inflammatory stimulus is resolved, negative regulators modulate the strength and duration of the activated MAPK signaling pathway and control the production of inflammatory cytokines, thus restraining the potentially devastating actions of the immunological system on the host and preventing self-destruction. These negative regulators include tyrosine, serine/threonine, and dual specificity phosphatases. A group of dual specificity protein phosphatases (often referred to as MAPK phosphatases (MKPs)) [69] are the primary phosphatases responsible for dephosphorylation/deactivation of MAP kinases (see Figure 2). MKP-1, an archetypal member of the MKP family, is essential for the dephosphorylation/deactivation of MAPKs p38 and JNK. Additionally, it serves as pivotal feedback control regulator in the innate immune response during microbial infection and thus, plays a significant role in the progression of periodontitis. 

### 2.2. Expression of Immune Cytokines

Periodontal diseases are characterized by chronic inflammation due to the overproduction of inflammatory mediators such as cytokines, chemokines, nitric oxide, and reactive oxygen species by immune and nonimmune cells. In the progression of the disease, the extent and severity of tissue destruction are the result of cytokine overproduction, which is largely dependent on the nature of the host-microbial interactions.

Cytokines interact functionally in networks and integrate aspects of innate and adaptive immunity, mobilizing leukocytes to the infection site, initiating the adaptive immune response, and initiating the acute phase response [16]. To date, two proinflammatory cytokines, TNF-*α* and IL-1*β*, are the best understood cytokines that correlate significantly with periodontal diseases. 

Of the IL-1 family, IL-1*α* and IL-1*β* have been recognized to be central proinflammatory cytokines with similar biological activity. IL-1ra is an endogenous receptor antagonist and an anti-inflammatory nonsignaling molecule that competes for receptor binding with IL-1*α* and IL-1*β*. The overall contribution of IL-1 to the proinflammatory response depends on the balance among these three molecules [70, 71]. In periodontal research, many studies are conducted to measure IL-1*β* in gingival tissue and gingival crevicular fluid (GCF) in patients with various periodontal conditions. Increased IL-1*β* expression has been consistently detected in both samples [72, 73] and was associated with periodontitis severity [74], and IL-1*β* decreased after treatment [75]. In periodontitis patients, IL-1*β* production increased via circulating monocytes or oral polymorphonuclear neutrophils (PMNs). Furthermore, blockage of IL-1*β* activity slowed progression of experimental periodontitis in primates [76]. In addition, IL-1*β* stimulates many cells to produce MMPs and prostaglandins, resulting in bone resorption and connective tissue degradation, all of which contribute to the pathogenesis of periodontitis [77].

TNF-*α* is released by activated monocytes, macrophages, and T-lymphocytes and promotes critical inflammatory responses. TNF-*α* induces bone resorption and upregulates PGE_2_ and MMP secretion during periodontal disease [56]. Clinical evidence indicates that patients with periodontal disease have increased TNF-*α* in the gingival crevice fluid [44], and TNF-*α* has been shown to be enhanced during destruction of periodontal tissues [49–51]. TNF-*α* binds to two distinct TNF receptors: TNF-R1 (p55) and TNF-R2 (p75), and most of TNF's inflammatory activities are transduced by TNF-R1. TNF-R2 is thought to enhance this activity by binding TNF and then passing it on to the TNF-R1 [78]. Blocking TNF-*α* has been proven to effectively inhibit osteoclast formation [79]. Indeed, the blockade of TNF can be used as a probe to understand the molecular basis of osteoclastogenesis and it may be a target for therapeutic agent development.

## 3. Cytokine Inhibition Strategies via MAPK Blockade

The contemporary concept of periodontal therapy focuses on eliminating bacteria through mechanical and chemotherapeutics means because the role of bacteria in the initiation and progress of periodontal disease is undisputed. Various therapeutic strategies aimed at the microorganisms, including local and systemic delivery of antimicrobial and antibiotic agents, have been studied over the years, but none of these methods has proven universally efficacious, particularly in the case of tissue-invasive species such as *Aggregatibacter actinomycetemcomitans *(*A. actinomycetemcomitans*). As we understand more about the immune response in the pathobiology of infectious diseases, including periodontitis, therapeutic strategies have focused more on host modulation. For example, therapeutic manipulation involving inhibition of TLR signaling using a small molecule inhibitor TAK-242 has been demonstrated to be beneficial to sepsis [80]. Specific soluble receptors (antagonists) of IL-1 and TNF-*α* inhibited the progression of bone loss by reducing the formation of osteoclast and recruitment of inflammatory cells in a nonhuman primate experimental periodontitis model [81]. Approaches to block the progression of inflammatory bone loss observed in periodontitis include host modulation of MMPs, COX2, and arachidonic acid metabolites. However, these therapies target singular mechanisms of alveolar bone destruction. Cytokines are well known to compensate for one another, thereby limiting the effect of cytokine-specific inhibitors. Alternatively, targeting a common regulatory mechanism for multiple cytokines may repress periodontal disease progression and improve treatment response. Also, inflammatory cell signaling pathways that generate inflammatory and tissue destruction proteins have become promising therapeutic targets. Therapeutic modulation of signaling pathways can affect various genes, depending not only on the pathway but also on the relative position targeted for inhibition in the signaling cascade. Because the MKK-MAPK-MK2 pathway phosphorylates downstream intermediates and regulates ARE proinflammatory cytokines, including IL-6, TNF-*α*, GM-CSF, IL-8, and iNOS, through mRNA stability, it and its pathway components could be excellent targets for therapeutic designs.

### 3.1. p38 Inhibition Studies

As described previously, p38 MAPK is an upstream effector common to many inflammatory cytokines. Activation of p38 MAPK signaling mediates inflammatory cytokine expressions such as IL-1*β*, IL-6, and TNF-*α* either directly or indirectly. These cytokines synergistically stimulate the production of other inflammatory cytokines, MMPs, and prostanoids [82–86]. p38 has also been implicated in the regulation of, IL-3, IL-8, macrophage inhibitory protein 1-*α* (MIP), GM-CSF, VEGF, urokinase-type plasminogen activator, and inducible NO synthase [87–90]. Evidence suggests that it is involved in rheumatoid arthritis, Alzheimer's disease, ischemic heart disease, asthma, dermatitis, inflammatory bowel disease, and periodontitis [86, 91–98].

Four members of the p38 MAPK family have been cloned: p38-*α*, -*β*, -*γ*, and -*δ*. All isoforms share conserved residues involved in ATP and ion binding and share significant sequence homology in their kinase domain and the 24- to 27 amino acid N-terminal to this domain. These regions are most likely to be involved in substrate specificity and activity. The *α* isoform is ubiquitously expressed to induce apoptosis, whereas the *β* isoforms is highly expressed in the brain and heart to promote cell survival in cardiac muscle cells. The *γ* isoform is chiefly expressed in muscle cells, and the *δ* isoform is expressed in the lung, kidney, gut, and salivary gland epithelium.

Bicyclic imidazole (compound SB203580) was initially identified to be an ATP-competitive inhibitor of p38 kinase [99], and later, more potent and specific inhibitors such as VX-745 and BIRB-796 were explored [100]. These developments lead to p38 kinase inhibition therapies which have been shown to be efficacious in several animal disease models, including rheumatoid arthritis (RA), psoriasis, Crohn's disease, stroke, asthma, chronic obstructive pulmonary disease (COPD), and periodontitis [99, 101–105]. For example, p38 inhibitors SC409 and SD282 have been shown to be efficacious in reducing and reversing bone and cartilage destruction in an experimental arthritis model [91]. In LPS-induced arthritis, mice treated with p38 MAPK inhibitors RO4399247 and AVE8677 diminished IL-6 to background levels [106]. Given that cytokines act synergistically, simultaneously blocking them is substantially more effective than blocking one alone. In the first study to test p38 inhibitors in humans, a single dose decreased TNF-*α*, IL-1, and -6 by 90%. Among inhibitors, an important distinction among isoforms is their ability to be inhibited by SB203580. Such inhibitors have 14.3 to >1,000 times greater activity against p38*α* than p38*β*, p38*γ*, or p38*δ* isoforms [91].

**Figure 3:**
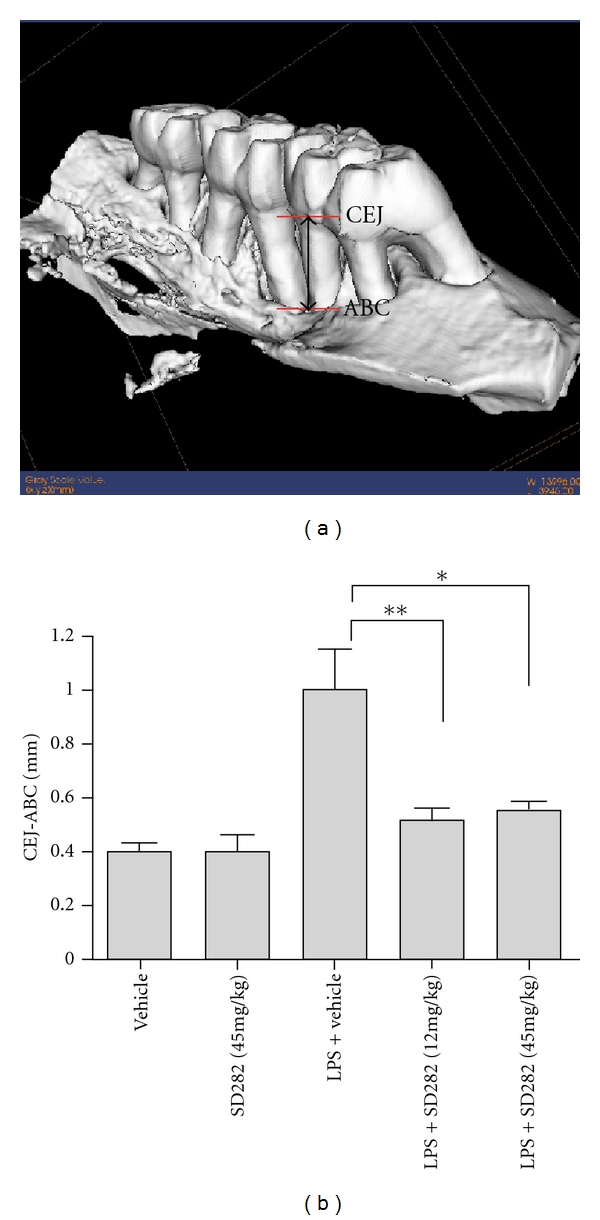
*A. actinomycetemcomitans* LPS induces significant linear bone loss which is blocked by SD-282. (a) Reformatted *μ*CT isoform display from 8 weeks *A. actinomycetemcomitans* LPS-injected rat maxillae exhibits dramatic palatal and interproximal bone loss. Landmarks used for linear measurements were the cementoenamel junction (CEJ) to the alveolar bone crest (ABC). Differences between these anatomical locations using defined locations of 2D displays determined alveolar bone loss. (b) Linear bone loss as measured from the CEF to AB (Mean ± SEM). Significant bone loss (*P* < 0.01) was observed between control (*n* = 6) and *A. actinomycetemcomitans* LPS injected rats (*n* = 12). Significant reduction of LPS-induced periodontal bone loss (**P < 0.01 for SD-282 [15 mg/kg; *n* = 8] and *P < 0.05 for SD-282 [45 mg/kg; *n* = 8]) [105]. Reproduced with permission.

Research from our laboratory documents the relevance of p38 MAPK in the regulation of expression of IL-6, MMP-13, and receptor activator of NF-*κ*B ligand in periodontally relevant resident cells, such as fibroblasts and osteoblasts, *in vitro *[17, 59, 107, 108]. *In vivo*, we have shown the significance of p38 MAPK signaling in periodontal disease progression, in which orally active p38 inhibitors reduced periopathogenic LPS-induced bone destruction in a rat model [105]. To study the preventive function of p38*α* inhibitors in periopathogenic LPS-induced experimental alveolar bone loss using a rat model, two simultaneous doses of SD-282 (15 or 45 mg/kg) were administered twice daily by oral gavage for 8 weeks. Bone area and volumetric analysis by *μ*CT indicated significant bone volume loss with LPS treatment, but this was blocked with both doses of p38*α* inhibitor (see Figure 3). Histological examination indicated significantly fewer tartrate-resistant acid phosphatase-positive osteoclasts adjacent to the areas of active bone resorption, including the periodontal ligament area, and a significant decrease in IL-6, IL-1*β*, and TNF-*α* expression in p38 inhibitor-treated groups compared with LPS groups by immunostaining. This proof-of-principle study supports—for the first time—the role of an orally active p38*α* MAPK inhibitor (SD-282) to potentially benefit LPS-induced alveolar bone loss. These data also suggest that the *α* isoform is the predominate isoform expressed in LPS-induced periodontal disease pathology.

Clinically, the therapeutic goal is to prevent further advancement of alveolar bone loss. Thus, in a second therapeutic model, we investigated the effect of a p38 MAPK inhibitor on established periodontal disease [109]. The periodontal disease state was established by LPS injections to the palatal molar gingiva three times per week for 4 weeks. p38*α* MAPK inhibitor SD282 (45 mg/kg) was administrated from weeks 5 through 8 via oral gavage in addition to LPS injections. The data from this study revealed that treatment with an orally active p38 MAPK inhibitor stopped the established periodontal disease progression *in vivo *and decreased inflammatory cytokine (IL-1*β*, TNF-*α*) expression and osteoclastogenesis. Interestingly, in this study, SD282 had a slight anabolic effect; the difference between the 8-week LPS-only and LPS + SD282 groups was significant for increased bone volume. The reasons for this are unclear and may be due to a relatively high suppression of osteoclastogenesis without compensatory cessation of osteoblastic differentiation. Conceptually, this makes p38 inhibitor strategies appealing as a host-modulating agent for treatment of periodontitis because physiologic bone turnover (induced by PTH/PTHrP) would occur, but inflammatory bone loss (induced by LPS, IL-1*β*, and TNF-*α*) would be pharmacologically antagonized.

Collectively, these data highlight the therapeutic potential of this novel class of inhibitors in bacterial-induced alveolar bone loss—the hallmark of periodontitis, but developing p38 inhibitors as a therapeutics in clinical settings have failed due to unacceptable safety profiles, central role for activation of various downstream kinases and transcription factors, ubiquitous expression, toxicity, significant off-target effects, and lack of oral bioavailability [110]. Preclinical and clinical side effects include hepatotoxicity, cardiotoxicity, light-headedness, central nervous system toxicities, skin rash, gastrointestinal tract symptoms, and bacterial infections [88, 111–115]. In addition, p38 MAPK-knockout mouse is embryonic lethal [90, 116–118]. Until now none of these p38 inhibitors has been approved for clinical usage.

### 3.2. Intraoral Silencing of MK2 Signaling

Due to the concerns about p38 inhibitors indicated above, targeting downstream substrates of p38 MAPK and factors that regulate transcription, nuclear export, mRNA stability, and translation could be a promising therapeutic alternative for inhibiting inflammatory gene expression to treat various inflammatory diseases. As a direct substrate of the stress-activated MAPK p38*α* and *β*[119], MAPK-activated protein kinase 2 (MAPKAPK-2, MK2) is regulated exclusively by p38*α*/*β* [119].

Landmark studies performed with MK2-deficient cells *in vitro *or with MK2-deficient mice *in vivo* clearly revealed the physiological roles of MK2 activation. The studies performed with MK2-deficient cells *in vitro* demonstrated a central role of MK2 in the production of proinflammatory mediators such as TNF-*α*, IL-1*β*, MIP-1*α*, IL-8, IL-6, and INF*γ* [120]. MK2 is also involved in atherogenesis by promoting the recruitment of inflammatory monocytes/macrophages through increasing endothelial expression of VCAM-1 and MCP-1 [121]. MK2-deficient mice show an increased stress resistance to LPS-induced endotoxic shock, owing to a severely impaired inflammatory response leading to a 90% reduction in TNF-*α* production, as well as reduction of IFN-*γ*, IL-1*β*, IL-6, and nitric oxide [120]. The MK2 gene deletion protected DBA/1LacJ mice from collagen-induced arthritis due to a significantly lower LPS-induced TNF-*α* and IL-6 serum levels when compared with wild-type controls [122]. Deficiency of MK2 also protected against cerebral ischemic injury [123]. In addition, MK2 also participates in other diverse cellular processes such as cell division, apoptosis, cell differentiation, endocytosis, reorganization of the cytoskeleton, cell migration, cell cycle progression, chromatin remodeling, respiratory burst, and chemotaxis [124–126].

Targeting MK2 should be a more specific target than p38, with potentially fewer side effects, because MK2 acts on a more limited downstream substrate repertoire compared to p38. Importantly, MK2-deficient mice are viable with a normal phenotype [90, 120]. Therefore, there has been much research exploiting MK2 as molecular target for the development of experimental therapeutics for number of conditions such as RA, Alzheimer's disease, atherosclerosis, and cancer. As periodontal disease has remarkably similar inflammatory pathways and mediator profiles with other inflammatory diseases [6, 127, 128], it is reasonable to anticipate that MK2 would be an attractive and potentially selective target for the treatment of periodontitis. However, targeting MK2 with small molecular inhibitors is complex and difficult because of the relatively planar ATP-binding site of this critical MAPK.

**Figure 4:**
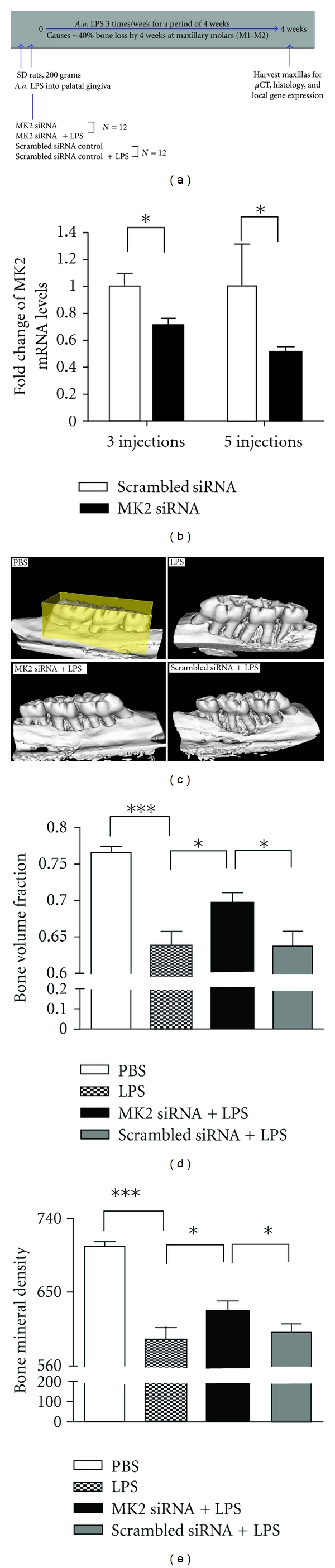
Specific MK2 siRNA *in vivo* delivery silenced target gene expression and reduced LPS-induced bone loss. (a) A schematic showing overall experimental protocol. (b) MK2 mRNA expression in palate gingiva after 3 and 5 times siRNA *in vivo* delivery. Results are expressed as mean ± SE (*n* = 5 or 6 rats/group, *P < 0.05). (c) Representative *μ*CT images of rat maxillae from indicated treatment groups. ROI for quantitative analysis is highlighted. (d) Volumetric analysis of bone loss levels. (e) Bone mineral density (BMD) analysis of bone loss levels. (*P < 0.05, ***P < 0.001) [153]. Reproduced with permission.

In our study, we hypothesized that silencing MK2 through an RNAi strategy would provide a novel anti-inflammatory target that selectively blocks signaling mechanisms needed for enhanced cytokine mRNA stability/translation in periodontitis progression. First, we validated MK2 silencing in cytokine production *in vitro* to evaluate the feasibility of choosing MK2, instead of p38, as a highly specific and potent drug candidate. Our data clearly showed that LPS-induced IL-6 expression was significantly attenuated, both at the mRNA and protein levels, a result consistent with previous observations in MK2^−/−^mice [120, 122]. We observed that MK2 siRNA delivery significantly reduced TNF-*α* mRNA and protein expression. The role of MK2 in the regulation of LPS-induced inflammatory cytokine gene expression is further confirmed by significant reductions of mRNA expression for COX-2, IL-1*β*, and the chemokine CXCL1 in cells transfected with MK2 siRNA. MK2 siRNA gene knockdown changed the activation of JNK and ERK MAPKs without obvious phospho-p38 expression variation, suggesting the existence of crosstalk and compensatory mechanisms and underscoring the complexities of Toll-like receptor signaling pathways. Secondly, *in vivo* studies employed the rat LPS-induced experimental periodontitis model to further elucidate the role of MK2 in the pathogenesis of periodontitis and evaluate the therapeutic potential by targeting MK2 employing an RNAi strategy in periodontal disease. The protection of MK2 siRNA from alveolar bone loss in LPS-induced periodontitis model was further verified by *μ*CT analysis (see Figure 4; [129]). Histological examination displayed that MK2 siRNA *in vivo* delivery attenuated the inflammatory infiltrate associated with *A. actinomycetemcomitans *LPS-induced bone loss. This is consistent with the decrease of osteoclast formation after MK2 silencing. In conclusion, with an RNAi strategy, our recent work validated that MK2 plays a role in a preventive model of experimental periodontitis, suggesting a novel target for controlling periodontal inflammation.

### 3.3. MKP-1 Alters MAPK Signaling in Periodontal Disease Progression

MAPKs are activated by phosphorylation of critical tyrosine, serine, or threonine residues. Negative regulation of MAPK activity is mediated by the MAPK phosphatases (MKPs) that dephosphorylate these functional residues [130]. To date, this group of dual-specificity protein phosphatases (MKPs) includes 11 members in mammalian cells and the founding member is MKP-1. MKP-1 is localized to the nucleus, where it preferentially dephosphorylates activated p38 and JNK compared with ERK MAPK [131]. *In vitro* studies conducted using cultured immortalized macrophages provided compelling evidence that MKP-1 attenuates TNF-*α* and IL-6 after LPS stimulation [132–134]. MKP-1 functions as a feedback control mechanism, which governs the production of proinflammatory cytokines by deactivating p38 and JNK, thereby limiting proinflammatory cytokine biosynthesis in innate immune cells exposed to microbial components [132, 133, 135]. Consistent with *in vitro* data, *MKP-1 *null mice had markedly more production of proinflammatory cytokine TNF-*α*, IL-6, and an anti-inflammatory cytokine IL-10 compared with wild-type animals. Sustained p38 and JNK activity in response to stress support the central role of MKP-1 in the restraint of the innate immune response and in the prevention of endotoxemia, experimentally induced autoimmune arthritis, septic shock syndrome, and multiorgan dysfunction during pathogenic microbial infection [134–138].

The critical role of the p38/MKP-1 axis of regulation on the innate immune response and in maintaining bone homeostasis has been clearly demonstrated. Moreover, as MKP-1 not only regulates p38 MAPK, but also JNK and ERK activities, overexpression of MKP-1 has potent capacity to prevent an exuberate immune response and osteoclastogenesis in response to stimuli compared with p38 MAPK inhibitors. Using gain- and loss-of-function approaches, the role and potential therapeutic target of MKP-1 in inflammatory bone loss was explored.

First, decreased IL-6 expression was observed in murine macrophage cell line RAW264.7 transfected with expression vector containing MKP-1 cDNA in pSRII-Flag. This data provided in macrophages supports the role of MKP-1 in negative regulation of *A. actinomycetemcomitans *LPS-induced p38 activation and IL-6 production [139]. MKP-1 plays a crucial role in decreasing inflammatory cytokine biosynthesis. Then in our chronic periodontitis model, wild-type and* MKP-1 *null mice received *A. actinomycetemcomitans *LPS injection in the palatal region or PBS control 3 times/wk for 30 days. Results indicated that, in LPS injected *MKP*-1^−/−^ mice, significantly greater bone loss occurred with more inflammatory infiltrate in the periodontal areas injected with LPS and a significant increase in osteoclastogenesis compared with *MKP*-1^−/−^ control sites or either wild-type littermates. MKP-1 displayed a protective response in this more chronic model of inflammation and bone loss [139]. 

**Figure 5:**
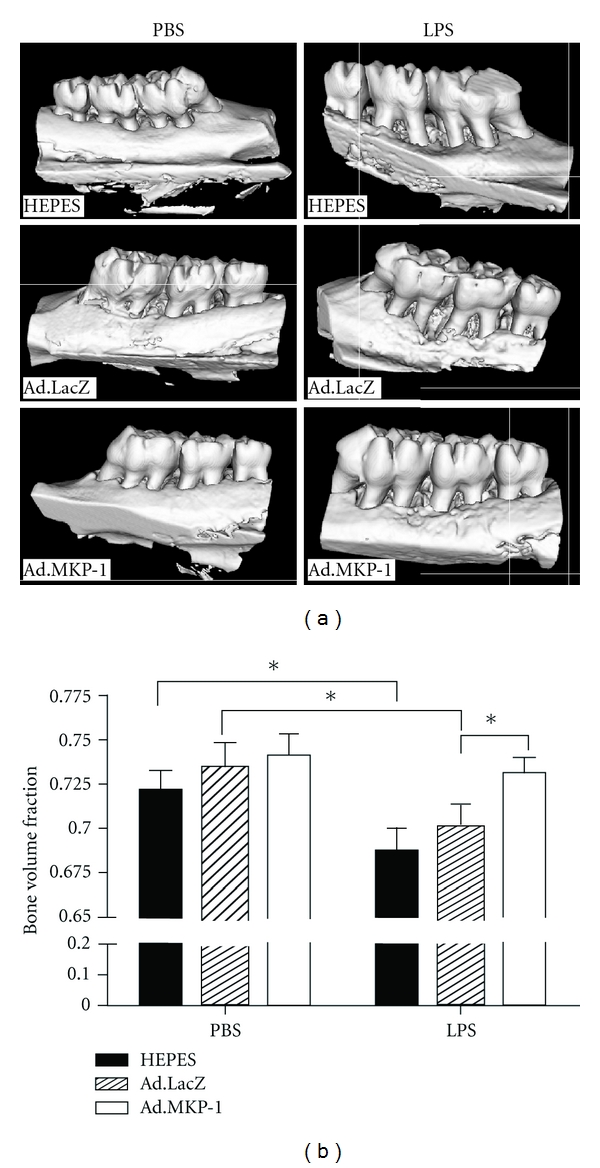
MKP-1 gene transfer alleviated bone resorption in rats after LPS challenge. Eight-week-old male Sprague-Dawley rats (17 rats/group) were injected either Ad.MKP-1, or Ad. LacZ (1×10^9^ pfu in 4 *μ*l), or HEPES buffered saline (4 *μ*l). Forty-eight hours after the adenovirus injection, the rats were injected with 2 *μ*l of either 20 *μ*g of LPS (from *A. actinomycetemcomitans*) or PBS three times a week for four weeks. (a) Representative microcomputed tomography images of rat maxillae from indicated treatment groups. (b) Volumetric analysis of bone loss levels (*n* = 7 for PBS groups, *n* = 10 for LPS groups, *P < 0.05); [140]. Reproduced with permission.

In gain-of-function experiments, MKP-1 was able to dephosphorylation all three MAPKs via MKP-1 gene transfer with recombinant adenovirus MKP-1 in rat macrophages. *Ex vivo *data indicated that MKP-1 gene transfer in bone marrow macrophages from MKP-1 KO mice significantly decreased IL-6, IL-10, TNF-*α*, and select chemokine levels compared with wild-type mice when stimulated by LPS [140]. In addition, bone marrow cultures from MKP-1 KO mice exhibited significantly more osteoclastogenesis induced by LPS than when compared with WT mice. This observation correlated with more osteoclasts seen in bone marrow cells of MKP-1 KO mice compared with osteoclasts from WT mice in response to LPS stimuli. Furthermore, *in vivo* MKP-1 gene transfer in an experimental periodontal disease model attenuated bone resorption induced by LPS (See Figure 5; [140]). Histological analysis confirmed that periodontal tissues transduced with Ad. MKP-1 exhibited less infiltrated inflammatory cells, less osteoclasts, and less IL-6 than compared with rats of control groups.

Together, our studies indicate the importance of MKP-1 in the development of immune responses that contribute to LPS-induced alveolar bone loss. It can be used as a key therapeutic target to control of inflammation-induced bone loss associated with increased MAPK activation.

### 3.4. Local Gene Delivery of TTP as an Anti-Inflammatory Agent

Cells of the immune system tightly regulate the production of potentially harmful cytokines at many levels: transcriptional, posttranscriptional, translational, and posttranslational levels. Posttranscriptional regulation of cytokines occurs at different stages such as nuclear export, cytoplasmic localization, and stability/degradation. Central to the posttranscriptional regulatory events is the interaction of RNA with RNA-binding proteins that influence their splicing, localization, stability, and association with the translation machinery [141–143].

The cisacting elements, which interacted with RNA-binding protein, are AU-rich elements (AREs) [67, 144]. They are located in the 3′ untranslated regions (UTRs) of many cytokines (e.g., granulocyte-macrophage colony-stimulating factor (GM-CSF), TNF-*α*, IL-2, IL-3, IL-6), and other proinflammatory factors (e.g., COX2 and MMP-13). A hallmark of AREs is the pentamer AUUUA that occurs either alone or clustered [145]. Recently, the structure and the functional significance of AREs have been clearly demonstrated by insertion strategy and deletion strategy.

At least 20 different proteins that can bind to ARE segments have been identified to date, including TTP, HuR, butyrate response factor (BRF)-1 and BRF-2, ARE/poly(U)-binding/degradation factor-1 (AUF-1), T-cell intracellular antigen-1 (TIA-1), and T-cell-restricted intracellular antigen-related protein (TIAR). However, only a subset of RNA-BPs has been shown to influence the stability or translational efficiency of target mRNAs. Some RNA-binding proteins, such as TTP, AU-binding factor 1, and K homology splicing-regulatory protein, promote mRNA decay, whereas others, like members of the Hu family, prevent mRNA degradation.

TTP is a well-characterized, zinc-finger-containing, RNA-binding protein. The function of TTP was elucidated through several studies with TTP-deficient mice. TTP deficiency is associated with cachexia, arthritis, autoimmunity, and myeloid hyperplasia, secondary to increased TNF-*α* and GM-CSF levels. In TTP^−/−^ mice, the increased cytokine production was shown to be a result of increased mRNA stability [66, 146]. Many studies also have demonstrated that the overexpression of TTP promoted the decay of reporter transcripts that contained AU-rich sequences from TNF-*α in vitro* [147].

TTP binds to AREs located in 3′ untranslated regions of cytokine genes and targets them to the exosome for rapid degradation. In this context, TTP can be considered an anti-inflammatory protein. As a point of therapeutic potential, we hypothesized that targeting a common upstream RNA-binding protein, TTP, may inhibit periodontitis progression.

**Figure 6:**
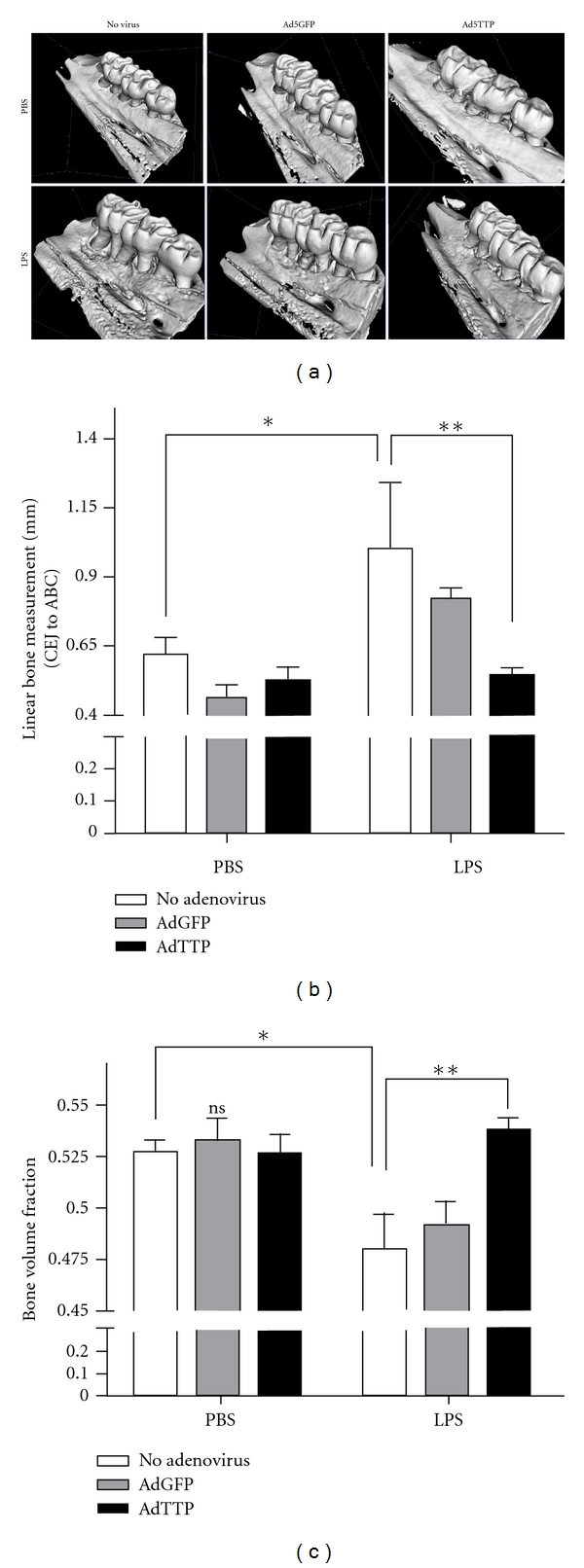
Ad5-TTP protects bone in experimental periodontal disease model. (a) representative microcomputed tomography images of rat maxillae from indicated treatment groups. (b) Linear measurement from cementoenamel junction (CEJ) to alveolar bone crest (ABC) (*n* = 8/group; *P < 0.05, **P < 0.01), (c) Volumetric analysis of bone loss levels (*n* = 8/group; *P < 0.05, **P < 0.01) [148]. Reproduced with permission.

Using adenoviral-delivered TTP, TTP overexpression was evaluated in a preventive model of experimental periodontitis to determine whether altering cytokine mRNA stability affected pathological bone resorption (see Figure 6; [148]). *In vivo *analysis indicated a significant protective effect from inflammation-induced bone loss and inflammatory infiltrate in animals overexpressing TTP compared with reporter controls. In addition, significant reductions of IL-6, TNF-*α*, and PGE_2_ were observed after TTP overexpression *in vitro *through a mechanism consistent with targeting mRNA stability. Collectively, these findings provide experimental evidence that mRNA stability is a valid therapeutic target in inflammatory bone loss. 

*In vitro*, we focused on testing if p38/MK2 signaling is required for cytokines expression regulation via TTP activity. Using MC3T3-E1 cell line as an osteoblastic model, we observed that p38 MAPK regulates IL-1*β*-stimulated IL-6 at a posttranscriptional mechanism and one of the primary targets of IL-6 gene regulation is the 3′ UTR of IL-6 [107]. Using mouse embryonic fibroblasts (MEFs) derived from p38*α* ^−/−^ mice, we further confirmed the above conclusion and determined p38*α* as the critical isoform. We identified three ARE elements that require p38*α* signaling, and IL-6 3′-UTR-(56–173) is critical for p38*α* to promote mRNA stability [149]. After this, the role of TTP in posttranscriptional regulation of IL-6 was elucidated. Genetic and siRNA-mediated knockdown of TTP resulted in increased IL-6 production and overexpression of TTP had the reverse effect [150]. Significant IL-6 mRNA expression and a long half-life were observed in TTP^−/−^ mice MEFs. Overexpression of TTP reduced IL-6 3′UTR luciferase reporter activity in an ARE-dependent manner. Mutation-based luciferase assays show that ARE2, ARE3, and ARE4 are required for TTP-mediated repression, and the constitutively activated p38-MK2 pathway abrogated TTP-mediated repression of IL-6 3′UTR reporter activity. An RNA immunoprecipitation assay indicated that the p38*α* deficiency resulted in increased affinity of TTP to IL-6 mRNA. Taken together, our serial studies showed that the RNA-binding protein, TTP, regulates IL-1*β*-induced IL-6 expression at the posttranscriptional level through an affinity shift for the transcript, which occurs in a p38 MAPK-dependent manner and involves specific AREs within the 3′UTR of the IL-6 mRNA.

## 4. Future Studies

Our research group has accumulated significant information that IL-6 regulation is highly dependent on p38 MAPK signaling in a variety of cell types [59, 105, 107, 108, 149, 151]. TTP directs mRNA stability of IL-6 largely in a p38 MAPK-dependent manner [150]. The clinical significance of this regulation was also demonstrated through inhibition of inflammation and bone loss via different strategies, such as small molecule inhibitors against p38 MAPK [105], MKP-1 overexpression, or TTP overexpression using gene transfer with recombinant adenovirus MKP-1 or TTP [140, 148, 149], and more recently using siRNA strategies targeting the p38 downstream kinase, MK2 [129]. Collectively, our series of studies targeting multiple molecules situated in inflammatory-related MAPK signaling cascade either to elucidate the functional mechanism or to verify the therapeutic potential for periodontal disease provide strong proof-of-principle evidence.

However, disease progression is complex with multiple aspects worthy of consideration. In human periodontal pathology, infectious bacteria are capable of interacting with the host. Although LPS is a potent inflammatory mediator, other components (e.g., cytoplasmic membranes, peptidoglycans, outer membrane proteins, capsules, and cell-surface fimbriae) within live organisms can induce apoptosis and modulate or evade the immune response. In addition, other classes of PRRs, such as Nod-like receptors (NLRs), have been recognized to have important roles in sensing intracellular infections [152]. NLRs have also been shown to modulate various signaling pathways, including p38 MAPK and NF-*κ*B, underscoring the complexity of TLR signaling and the crosstalk with other signaling pathways involved in the pathobiology of periodontal disease. Cytokines in the periodontium integrate aspects of innate and adaptive immunity. It is becoming clear, however, that cytokines do not function in isolation, but form complex interactive networks involving both pro- and anti-inflammatory effects.

Future studies are being planned with large animals or nonhuman primates to further address the therapeutic effect of small molecule inhibitor strategies. In addition, elucidation of fundamental mechanisms, finding powerful target candidates, developing clinically viable delivery systems, and optimizing dose and delivery routes must be addressed, along with exploration of different infection models to elucidate the potential of these strategies to arrest other inflammatory diseases.
